# CenH3/CID Incorporation Is Not Dependent on the Chromatin Assembly Factor CHD1 in *Drosophila*

**DOI:** 10.1371/journal.pone.0010120

**Published:** 2010-04-09

**Authors:** Valerie Podhraski, Beatriz Campo-Fernandez, Hildegard Wörle, Paolo Piatti, Harald Niederegger, Günther Böck, Dmitry V. Fyodorov, Alexandra Lusser

**Affiliations:** 1 Division of Molecular Biology, Biocenter, Innsbruck Medical University, Innsbruck, Austria; 2 Tyrolean Cancer Research Institute, Innsbruck, Austria; 3 Division of Experimental Pathophysiology and Immunology, Biocenter, Innsbruck Medical University, Innsbruck, Austria; 4 Department of Cell Biology, Albert-Einstein College of Medicine, New York, New York, United States of America; Duke University, United States of America

## Abstract

CHD1 is a SNF2-related ATPase that is required for the genome-wide incorporation of variant histone H3.3 in the paternal pronucleus as well as in transcriptionally active nuclei in *Drosophila* embryos. The *S. pombe* and vertebrate orthologs of CHD1 have been implicated in the assembly of the centromeric histone H3 variant CenH3^CENP-A^, which occurs in a DNA replication-independent manner. Here, we examined whether CHD1 participates in the assembly of CenH3^CID^ in *Drosophila*. In contrast to the findings in fission yeast and vertebrate cells, our evidence clearly argues against such a role for CHD1 in *Drosophila*. CHD1 does not localize to centromeres in either S2 cells or developing fly embryos. Down-regulation of CHD1 in S2 cells by RNAi reveals unchanged levels of CenH3^CID^ at the centromeres. Most notably, ablation of functional CHD1 in *Chd1* mutant fly embryos does not interfere with centromere and kinetochore assembly, as the levels and localization of CenH3^CID^, CENP-C and BubR1 in the mutant embryos remain similar to those seen in wild-type embryos. These results indicate that *Drosophila* CHD1 has no direct function in the incorporation of the centromeric H3 variant CenH3^CID^ into chromatin. Therefore, centromeric chromatin assembly may involve different mechanisms in different organisms.

## Introduction

The incorporation of variants of the histones H3 and H2A, such as H3.3, CenH3 or H2A.Z, into chromatin correlates with functional specification of genomic regions and is thought to contribute to the epigenetic memory of a cell [1]. In contrast to canonical histones, which are assembled during DNA replication, histone variants are incorporated into chromatin throughout the cell cycle. However, a thorough understanding of the mechanisms of replication-independent assembly of histone variants remains to be established.

*In vitro*, the concerted action of histone chaperones and ATP-utilizing factors is required for histone deposition and nucleosome arrangement [2], [3]. The ATP-dependent motor protein *Drosophila* CHD1 mediates the reconstitution of periodic nucleosome arrays in conjunction with the histone chaperone NAP1 in an *in vitro* chromatin assembly system [4]. We have recently shown that *in vivo*, CHD1 is required for the replication and transcription-independent genome-wide assembly of the variant histone H3.3 in the paternal pronucleus and in transcriptionally active nuclei during embryonic development [5], thus confirming its role as a chromatin assembly factor.

Another H3 variant that is incorporated into chromatin in a replication-independent manner is the centromere-specific histone CenH3 (also known as CENP-A, CID, Cnp1, Cse4). Centromeres are specialized regions within eukaryotic chromatin that direct the faithful segregation of chromosomes during mitosis by serving as an assembly platform for the kinetochore. Notably, centromeric DNA sequence composition is not conserved among organisms, and it is therefore commonly thought that epigenetic mechanisms determine centromere identity and function [6], [7]. A distinguishing feature of centromeric chromatin in all organisms is the presence of CenH3-containing nucleosomes [1], [8]. CenH3^CENP-A^ incorporation occurs during late mitosis and G1 in human cells [9]. In cleavage-stage *Drosophila* embryos, in which the cell cycle lacks gap phases [10], the assembly of CenH3^CID^ into centromeric chromatin takes place during anaphase [11].

Two studies have implicated CHD1 in the formation of centromeric chromatin. In fission yeast, deletion of the CHD1 ortholog *Hrp1* was found to result in decreased incorporation of CenH3^Cnp1^ [12]. More recently, it was reported that CHD1 localizes to centromeres throughout the cell cycle in chicken DT40 and in HeLa cells. Furthermore, RNAi-mediated knockdown of CHD1 led to a loss of CenH3^CENP-A^ signal intensities at centromeres suggesting that CHD1 is required for the incorporation of CenH3^CENP-A^ [13].

In this study, we examined the function of CHD1 in centromeric chromatin assembly in *Drosophila*. In contrast to findings in fission yeast and vertebrate cells, we observed that *Drosophila* CHD1 is not required for CenH3^CID^ incorporation and kinetochore integrity.

## Results

### Dynamic localization pattern of CHD1 in *Drosophila* cells

**Figure 1:**
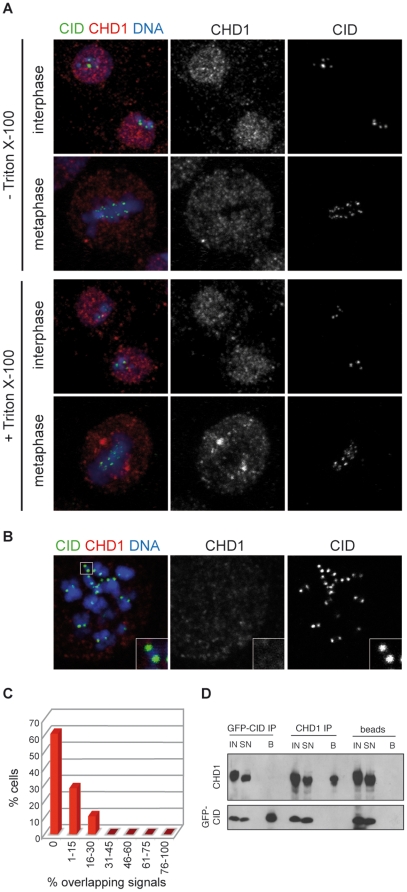
CHD1 is not present at centromeres in *Drosophila* S2 cells. A) CHD1 displays nuclear staining during interphase and redistributes to the cytoplasm during mitosis. S2 cells stably expressing EGFP-tagged CenH3^CID^ were treated (bottom) or not (top) with Triton X-100 before fixation to reduce the amount of soluble protein. Cells were stained with anti-CHD1 (red) and anti-GFP (green) antibodies. DNA is shown in blue. B) CHD1 is absent from chromosomes at metaphase. Spreads of metaphase chromosomes from EGFP-CenH3^CID^-expressing S2 cells were stained with antibodies against CHD1 (red) and GFP (green). C) Quantification of overlapping CHD1 and EGFP-CenH3^CID^ signals. Percentages of overlapping signals per cell were calculated and plotted against the percentage of cells displaying similar ratios of overlap. D) CHD1 and EGFP-CenH3^CID^ do not interact. Co-immunoprecipitations were performed on micrococcal nuclease treated S2 cell extracts with antibodies against GFP, CHD1 or protein A sepharose beads only. Aliquots of the input (IN) fraction, supernatant (SN) and eluted beads (B) were subjected to immunoblotting with anti-CID and anti-CHD1 antibodies.

Previous reports have demonstrated that CHD1 resides in the nucleus in mammalian cells during interphase but is released into the cytoplasm during mitosis [14], [15]. Recently, CHD1 was shown to be present at centromeres throughout the cell cycle in chicken and human cells [13]. To investigate the role of *Drosophila* CHD1 in CenH3^CID^ incorporation into chromatin, we examined the potential colocalization of CHD1 with CenH3^CID^ at centromeres in *Drosophila* S2 cells. To this end, we established a stable S2 cell line that allows for inducible expression of EGFP-tagged CenH3^CID^. Depending on the amount of overexpressed protein, EGFP-CenH3^CID^ is incorporated into authentic centromeres but may also form additional ectopic centromeres [16]. By using an antibody against the C-terminal portion of *Drosophila* CHD1 [17] we observed a granular staining pattern of interphase nuclei in S2 cells and the release of the protein from chromatin into the cytoplasm during mitosis (Figure 1A). In the majority of cells (61%; n = 54) we did not detect overlaps of CHD1 and CenH3^CID^ immunosignals in interphase or during mitosis (Figure 1A and C). Occasionally, merging signals were observed in some interphase nuclei. Quantification of these signals, however, revealed no conspicuous accumulation of overlapping signals in certain populations of cells, which might suggest cell cycle-dependent colocalization of CHD1 and EGFP-CenH3^CID^ (Figure 1C). Moreover, out of 447 CenH3^CID^ foci evaluated only 31 (6.9%) showed (partial) overlaps with CHD1 signals. Similar results were obtained when cells were treated with 0.1% Triton X-100 before fixation to reduce the amount of CHD1 that is not bound to chromatin (Figure 1A). In support of these observations, no overlapping signals of anti-EGFP-CenH3^CID^ and anti-CHD1 staining were detected on mitotic chromosome spreads of S2 cells (Figure 1B).

These results are clearly different from the observed colocalization of CHD1 and CenH3^CENP-A^ in vertebrate cells [13]. To rule out the possibility that our antibody does not recognize CHD1 when it is centromere-bound, we performed the same experiments with an antibody raised against two CHD1 peptides (Figure S1A and S2A). Moreover, we examined the localization pattern of CHD1 in an S2 cell line stably expressing Flag-tagged CHD1 (Figure S1D) by staining with anti-Flag antibodies (Figure S2B). Both approaches confirmed the absence of CHD1 at centromeric chromatin regions (Figure S2A and S2B).

We also examined whether there is a physical interaction between CHD1 and EGFP-CenH3^CID^ in S2 cells. To this end, chromatin from S2 cells was extensively digested with micrococcal nuclease to preferentially release mononucleosomes followed by immunoprecipitation with either anti-GFP or anti-CHD1 antibodies. Subsequent western blot analysis failed to reveal mutual co-precipitation of the two proteins (Figure 1D). Thus, in cultured *Drosophila* cells there is neither a colocalization of CHD1 and CenH3^CID^ nor do these proteins show direct or indirect physical interaction.

**Figure 2:**
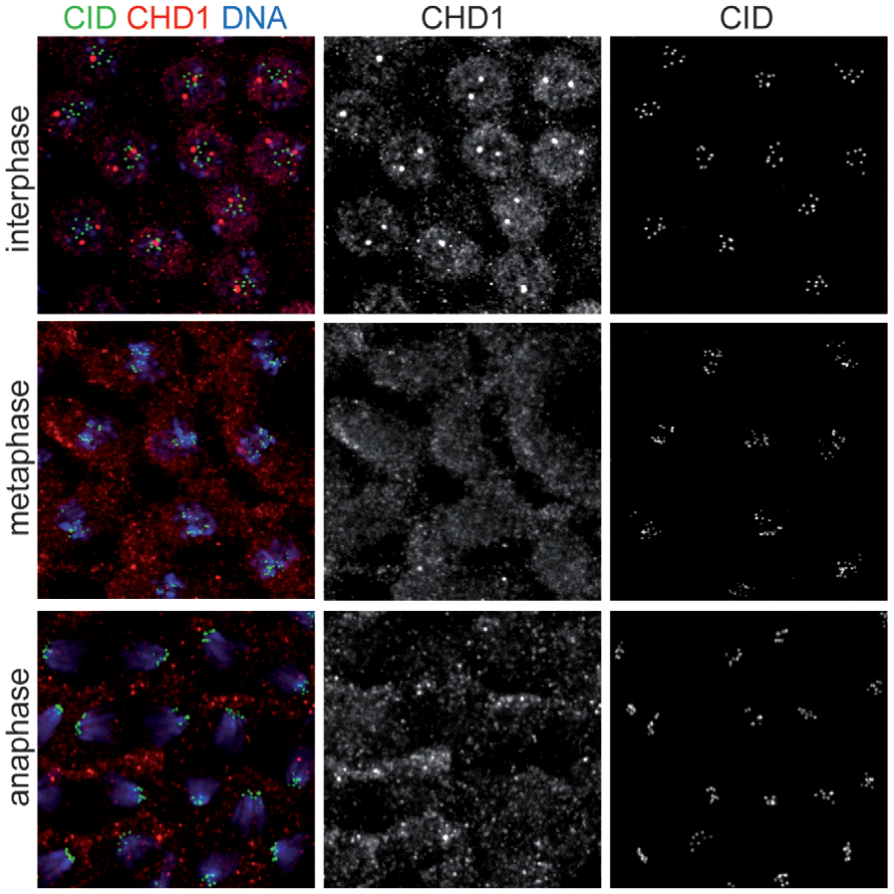
CHD1 and CenH3^CID^ do not colocalize in *Drosophila* embryos. Embryos laid by transgenic female flies expressing EGFP-tagged CenH3^CID^ [11] were collected at 0–2 h after egg deposition and stained with antibodies against CHD1 (red) and GFP (green) to detect EGFP-CenH3^CID^. DNA was stained with DAPI (blue). Three syncytial embryos with interphase, metaphase and anaphase nuclei, respectively, are shown. CHD1 does not colocalize with centromeres at any cell cycle stage.

Next we examined whether CHD1 colocalizes with CenH3^CID^ *in vivo*. Embryos from flies expressing an EGFP-tagged version of CenH3^CID^ [11] were collected at 0–2 h after egg deposition and analyzed. CHD1 did not colocalize with the centromeres at any cell cycle stage in syncytial *Drosophila* embryos (Figure 2 and data not shown). Hence, we conclude that there are clear differences in the intranuclear localization of CHD1 between vertebrate and *Drosophila* cells.

### CHD1 depletion does not affect cell cycle progression and CenH3^CID^ loading in S2 cells

**Figure 3:**
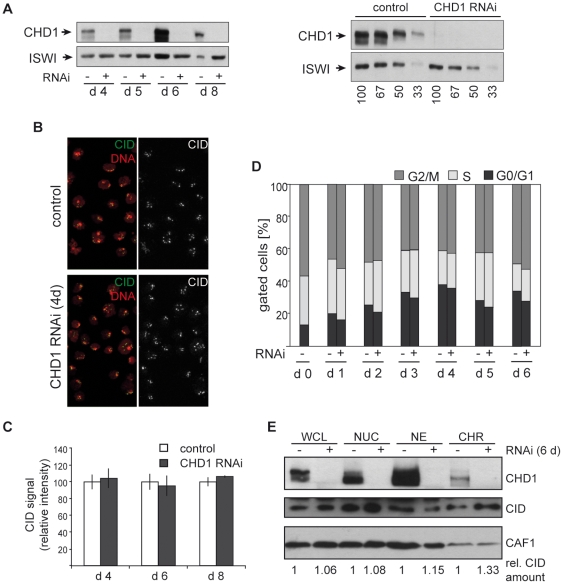
Cell cycle progression and CenH3^CID^ loading are not affected by RNAi-mediated downregulation of CHD1. A) Treatment of S2 cells with dsRNA targeting CHD1 for 4, 5, 6 and 8 days results in a decrease of CHD1 protein below detection limits. To control for equal loading western blots were incubated with an antibody against *Drosophila* ISWI (bottom). A serial dilution of control extracts was used to quantify CHD1 knockdown (right panel). B) CHD1 RNAi treated (4 days) and control cells were stained with anti-CenH3^CID^ antibodies (green). DNA was counterstained with DAPI (red). C) Signal intensities of centromeric foci after staining with anti-CenH3^CID^ antibodies were quantified as described in Materials and Methods. Error bars denote standard deviations of signals obtained from the following numbers of interphase nuclei: day 4 control and RNAi, n = 29; day 6 control, n = 168; day 6 RNAi, n = 148; day 8 control, n = 208; day 8 RNAi, n = 169. Signal intensities remained unchanged despite extensive CHD1 depletion. D) Cell cycle profiles of CHD1 RNAi treated (+) and untreated (−) control cells were determined by FACS analysis at different times (days 0–6) of dsRNA incubation. No significant changes in cell cycle progression patterns are detectable. E) Immunoblot analysis of cellular fractions of control and CHD1 RNAi treated cells with antibodies against CHD1 and CID. CAF-1 p55 was used as a loading control. WCL, whole cell lysate; NUC, nuclei; NE, high salt nuclear extract; CHR, chromatin core fraction (high salt insoluble fraction). The relative amounts of CID in the different fractions remain largely unaltered after CHD1-depletion.

The localization pattern of CHD1 might not necessarily reflect its cellular functions. Thus, low levels of CHD1 that escape detection by immunofluorescence might be sufficient to allow functional participation of CHD1 in the CenH3^CID^ loading process. To investigate a potential role of CHD1 in the incorporation of CenH3^CID^ into centromeric chromatin, we performed RNAi-mediated knockdown of CHD1 in S2 cells. Treatment of S2 cells with a combination of two CHD1 dsRNA probes resulted in the reduction of CHD1 protein expression below the detection limit (Figures 3A and S1C). We then examined the occurrence of aberrant mitotic figures and/or missegregation of chromosomes in S2 cells after various times of RNAi treatment (2 days – 8 days). Such effects should be expected, if CHD1 affects CenH3^CID^ loading and subsequent kinetochore formation [18], [19]. However, the rare incidence of mitotic defects remained unchanged in CHD1-depleted cells relative to untreated cells (data not shown). We also analyzed the cell cycle profiles of RNAi-treated and untreated cells by FACS analysis, but did not detect any significant alterations in the distribution of the different cell cycle stages (Figure 3D). Likewise, staining of CHD1-depleted cells with anti-CenH3^CID^ antibodies and quantification of the intensity of CenH3^CID^ fluorescence signals revealed no reduction in CenH3^CID^ levels in cells after 4, 6 or 8 days of RNAi treatment relative to control cells (Figures 3B and C). In contrast, depletion of the inner kinetochore component CENP-C, which had been demonstrated before to be necessary for CenH3^CID^ loading [20], caused defects in nuclear morphology (Figures S3A and B) and ∼60% reduction in CenH3^CID^ levels relative to control cells after 4 days of RNAi treatment (Figure S3C). By day 6 of RNAi treatment most of the cells had died (data not shown).

Despite the fact that CenH3^CID^ levels at the centromeres were not altered by CHD1 depletion, it was possible that the association of CenH3^CID^ with the chromatin was perturbed in a way similar to what has recently been reported for human CenH3^CENP-A^ upon depletion of the chromatin remodeling complex RSF [21]. To examine this idea, whole cell lysate, nuclear extracts and chromatin core fractions were prepared from CHD1-RNAi and control cells and CenH3^CID^ levels were quantified on western blots. These analyses did not show increased solubility of CenH3^CID^ in CHD1-depleted versus control cells. In all cellular fractions CenH3^CID^ levels were similar in RNAi-treated and control cells (Figure 3E).

Collectively, these data argue against a functional role for CHD1 in the deposition or maintenance of CenH3^CID^ at centromeres in *Drosophila* cells. It was possible, however, that small amounts of CHD1 that were not removed by RNAi-mediated knockdown prevented the detection of centromeric chromatin assembly defects. Hence, we investigated centromeric chromatin formation in *Chd1* null mutant fly embryos.

### CenH3^CID^ loading is not compromised in *Chd1* mutant *Drosophila* embryos

**Figure 4:**
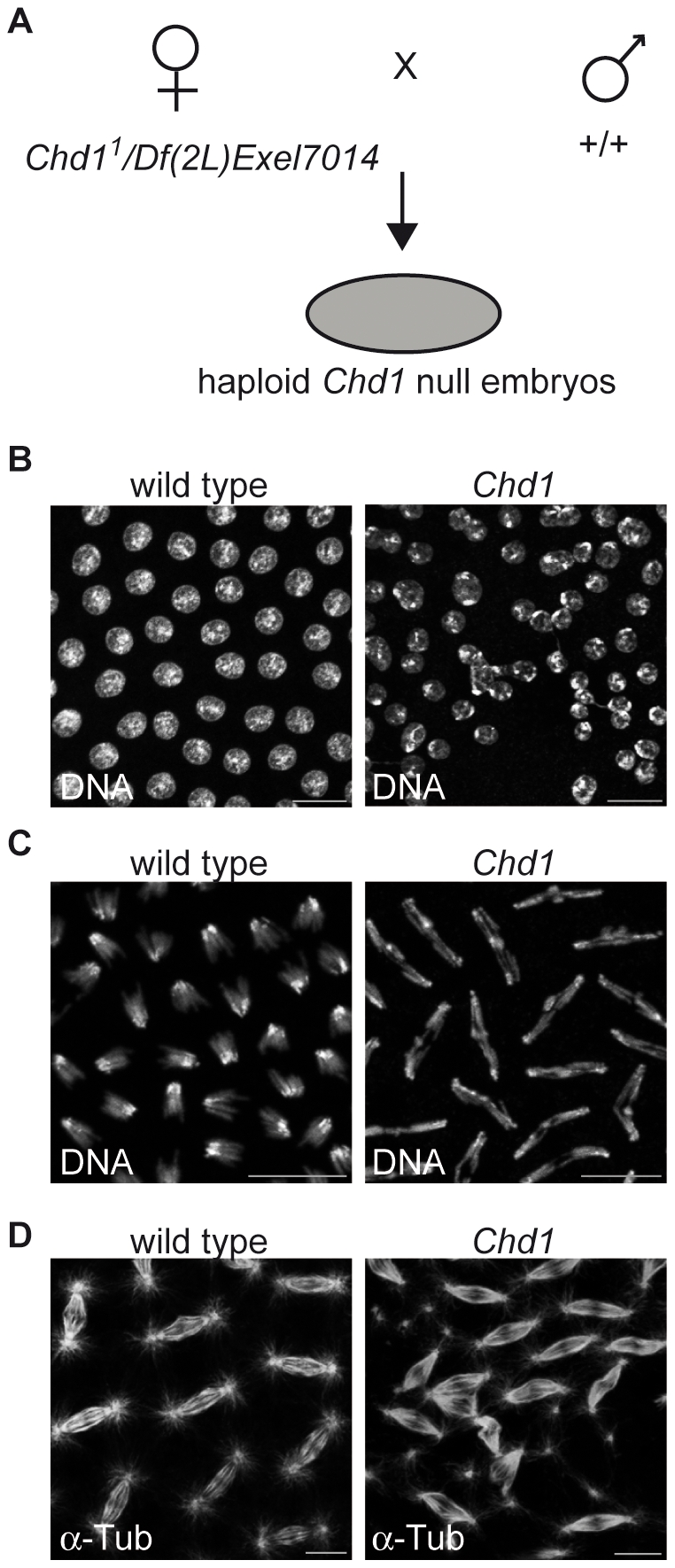
A fraction of *Drosophila Chd1* null embryos displays aberrant nuclear morphology and mitotic defects. A) Schematic representation of the generation of *Chd1* null embryos. B) The *Chd1* mutation occasionally leads to the appearance of irregularly sized and arranged nuclei. C) Mitotic defects, such as lagging chromosomes and disorganized spindles (D), were observed in 15.6% of mutant embryos (n = 295) in contrast to only 1% (n = 209) of wild-type embryos. DNA was stained with DAPI (B, C), mitotic spindles were visualized by staining with an antibody against α-tubulin (D). Scale bars  = 10 µm.

**Figure 5:**
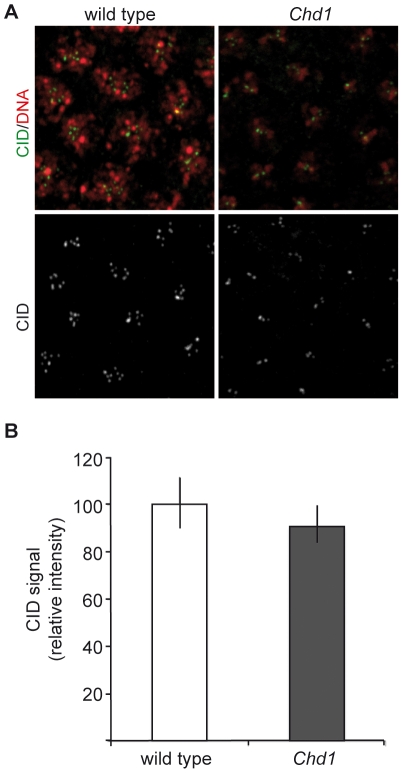
CenH3^CID^ loading is not compromised in *Chd1* null embryos. A) Embryos from wild-type and *Chd1* null flies were stained with anti-CenH3^CID^ antibodies (green). DNA was visualized with DAPI (red). B) Quantification of signal intensities of CenH3^CID^ foci in wild-type and *Chd1* null embryos (5 embryos each) as described in Material and Methods. CenH3^CID^ signals have similar intensities indicating that CHD1 has no impact on CenH3^CID^ loading.

We had previously shown that embryos deposited by *Chd1* null mutant females (*Df(2L)Chd1^1^/Df(2L)Exel7014*) are not viable [5]. The majority of these embryos are arrested at the earliest stages of development due to their inability to incorporate H3.3 into paternal chromatin in the absence of CHD1. Some embryos, however, survive until the syncytial blastoderm and even early gastrulation stages with haploid, maternally-derived chromosome content [5]. These embryos are completely devoid of CHD1, since the mothers do not produce CHD1 and the paternal chromosomes are lost at fertilization. We used these embryos to determine whether CHD1 is required for CenH3^CID^ incorporation during early embryonic development. DNA staining of syncytial embryos deposited by *Df(2L)Chd1^1^/Df(2L)Exel7014* females (for simplicity termed *Chd1* null embryos; Figure 4A) revealed mitotic defects, such as chromosome missegregation, lagging chromosomes or aberrant spindle appearance, in 15.6% of the mutant embryos (n = 295; Figure 4B, C, D) compared to 1% (n = 209) of wild-type embryos. A small fraction (1.7%) of the mutant embryos also displayed micro- and macronuclei as well as irregular arrangements of nuclei (Figure 4B). Although these defects could potentially be due to compromised centromere function, staining of the embryos with anti-CenH3^CID^ antibodies did not reveal aberrant localization or a reduction in CenH3^CID^ signal intensities (Figure 5A and B). It is important to note that *Chd1*-deficient embryos contain only 4 chromosomes corresponding to the maternal genome. This is reflected by the appearance of fewer centromeric foci than in wild-type embryos, which have 8 chromosomes per nucleus. Consistent with our observations in S2 cells, these data argue that CHD1 does not have an essential function in centromeric chromatin assembly.

### Intact localization of kinetochore components in *Chd1* null embryos

**Figure 6:**
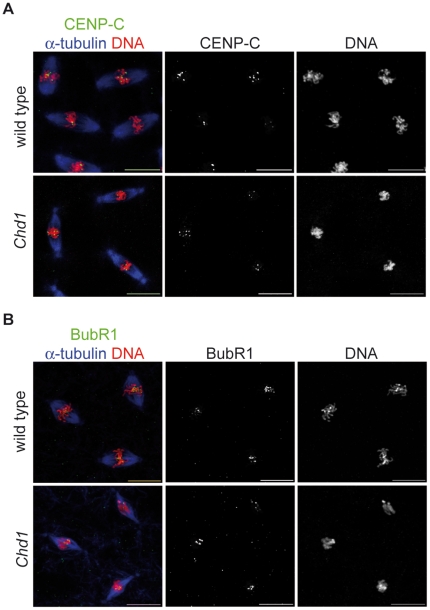
Recruitment of kinetochore components is not disturbed in the absence of CHD1. Wild-type and *Chd1* null embryos were stained with antibodies against α-tubulin (blue) and either the inner kinetochore protein CENP-C (A) or the outer kinetochore protein BubR1 (B; both in green). DNA was counterstained with propidium iodide (red). Neither CENP-C nor BubR1 show apparent localization defects in the absence of CHD1. Scale bars  = 10 µm.

The presence of normal CenH3^CID^ amounts at centromeres in *Chd1* null embryos strongly suggests that CHD1 is not necessary for CenH3^CID^ loading onto the DNA. However, CHD1 might still be involved in the remodeling of centromeric nucleosomes in order to allow proper kinetochore assembly. Since we have observed nuclear defects in *Chd1* null embryos (see above) that could potentially be due to compromised kinetochores, we examined the effect of the absence of CHD1 upon the localization of the inner kinetochore component CENP-C [18] as well as of the outer kinetochore component BubRI [22]. Stainings of wild-type and *Chd1* null embryos with antibodies against these proteins revealed that both proteins were correctly localized to centromeres (Figure 6A and B).

Thus, the formation of functional kinetochores appears to be unaffected in the absence of CHD1. These data further support the conclusion that CHD1 does not contribute in a critical manner to the functions of CenH3^CID^ chromatin in *Drosophila*.

## Discussion

Our analysis of the role of the ATP-dependent chromatin assembly factor CHD1 in centromeric chromatin formation revealed that *Drosophila* CHD1 is unlikely to play an important part in the assembly and/or maintenance of CenH3^CID^ at the centromeres. We provide multiple lines of evidence, including a lack of centromeric localization of CHD1, the absence of CenH3^CID^ loading defects in RNAi-treated cells as well as in *Chd1* mutant fly embryos and the intact localization of the kinetochore components CENP-C and BubRI in *Chd1* null embryos, that strongly argue against a critical function for CHD1 in this process. These findings stand in contrast to those from previous studies of CHD1 in *S. pombe*, chicken and human cells [12], [13]. In *hrp1Δ* mutant strains in fission yeast, it was shown by ChIP that CenH3^Cnp1^ levels are substantially reduced, whereas H3 levels are increased relative to wild-type yeast over the central core region of the centromeres. Moreover, Hrp1 was found to colocalize with centromeric regions during G1/S phase [12]. Centromeric localization of CHD1 was also observed in chicken DT40 cells, and RNAi-mediated depletion of CHD1 in human cells resulted in a strong decrease in CenH3^CENP-A^ centromeric signals [13]. The reasons for the observed differences between these and our data are unlikely to be technical in nature. Our results were obtained by multiple distinct approaches, all of which led to the conclusion that CHD1 does not play a critical role in CenH3^CID^ incorporation in *Drosophila*.

One explanation for that might be that centromeric chromatin assembly in different organisms might involve distinct mechanisms. This idea is consistent with the high degree of diversity of centromere organization and kinetochore components among different species [6]. Moreover, the structure of CenH3 itself differs considerably between species. Gene evolution studies revealed that the *Drosophila* and *Arabidopsis* CenH3 genes are subject to adaptive evolution, particularly in the regions that encode the DNA-interacting Loop 1 [23]. In contrast, no positive selection was observed in the evolution of mammalian or grass CenH3 [24]. The targeting of *Drosophila* CenH3^CID^ to centromeric regions is dependent on the Loop 1 region, and heterologously expressed CenH3 proteins from yeast and mammals did not localize to centromeres in *Drosophila* [25]. On the other hand, it was shown that budding yeast CenH3^Cse4^ could structurally and functionally substitute for human CenH3^CENP-A^ [26]. Hence, species-specific structural differences in the CenH3 proteins, such as the long N-terminal tail region that is unique to *Drosophila* CenH3^CID^, might result in the use of different assembly machineries. It is interesting to note in this context that the human CenH3^CENP-A^-specific chaperone HJURP, which was recently identified to bind directly to CenH3^CENP-A^ and to be required for CenH3^CENP-A^ incorporation into chromatin [27], [28], appears to lack orthologs in *Drosophila* and *Caenorhabditis* but shares similarities with the fungal CenH3^Cnp1^-specific chaperone Scm3 [29]. In *Drosophila*, the H3.3 specific chaperone HIRA was found to be required for the assembly of the histone variant H3.3 into paternal pronuclear chromatin [30]. We discovered a similar requirement for CHD1, suggesting that CHD1 and HIRA could cooperate in the H3.3 assembly pathway [5]. In an analogous manner, it is possible that CHD1 and HJURP might cooperate in the assembly of CenH3^CENP-A^ in vertebrates and fungi. In *Drosophila*, however, where there might be no HJURP, CHD1 might have lost a function in centromeric chromatin assembly and other factor(s) might have taken over its role. Intriguingly, a recent screen for CID localization-deficient genes in *Drosophila* identified CAL1 and CENP-C to be necessary for CenH3^CID^ assembly at the centromere [20]. The CAL1 protein is well conserved within *Drosophilids* but appears to lack homologues in other species. Although the exact role of CAL1 in centromeric chromatin assembly remains unclear, it was shown that CAL1 interacts with CenH3^CID^ and CENP-C in mononucleosomal chromatin fractions. Moreover, loss of CAL1 prevented centromeric localization of newly synthesized CenH3^CID^ and resulted in delocalization of CENP-C [20]. The hSNF2-containing ATP-dependent remodeling factor RSF was recently reported to interact with CenH3^CENP-A^ mononucleosomes in human cells and to play a role in the incorporation of CenH3^CENP-A^ [21]. In *Drosophila*, RSF has been implicated in the incorporation of the histone H2A variant H2Av. Defects in centromeric chromatin formation have not been observed in *dRsf1* mutant flies [31]. It is possible, however, that robust compensation mechanisms in *Drosophila* so far have hindered the identification of molecular motor proteins dedicated to the assembly of centromeric nucleosomes. Future studies using combined knock-down of different remodeling factors with histone chaperones, such as CAF-1 p55 [32], might therefore contribute to the elucidation of the role of ATP-dependent motor proteins in centromeric chromatin formation.

## Materials and Methods

### Fly strains

To obtain *Chd1* null embryos *Df(2L)Chd1^1^/Df(2L)Exel7014* virgin females were mated to wild type males as described [5]. Embryos were collected at 0–1 h or 0–2 h after egg deposition at 25°C and immediately processed for immunofluorescence staining.

### Generation of a stable EGFP-CID cell line

The coding sequence of EGFP was cloned into *Nhe*I*/Sac*II digested vector pMT-LacZ (Invitrogen) yielding vector pMT-EGFP. Since pMT-LacZ has no *Nhe*I site, this site had been generated by inserting a double stranded oligonucleotide containing an *Nhe*I site into the *Kpn*I/*Spe*I sites of the vector. The CID coding region was amplified by PCR from genomic DNA derived from wild-type flies and cloned into *Bgl*II/*Sac*II digested vector pMT-EGFP in frame with EGFP to give an N-terminal fusion product of EGFP with CID (pMT-EGFP-CID). To generate stably transformed cell lines, *Drosophila* S2 cells [33] were co-transfected with the pMT-EGFP-CID expression vector and pCo-Hygro (Invitrogen) using Effectene transfection reagent (Qiagen). Stably transfected cells were selected in the presence of 0.75 mg/ml hygromycin B in Schneider's *Drosophila* medium (Lonza). EGFP-CID expression was induced by addition of CuSO_4_ to a final concentration of 750 µM in growth medium for 24 h and monitored by western blot analysis of total cell lysates. Low amounts of EGFP-CID expression were obtained in uninduced cells due to some leakiness of the promoter.

### dsRNA treatment of S2 cells

To generate dsRNA probes for RNAi, DNA fragments corresponding to nt 70–599 (529 bp) and nt 1878–2306 (429 bp) of *Drosophila* CHD1 mRNA and to nt 832–1319 (488 bp) and nt 3684–4178 (495 bp) of *Drosophila* CENP-C mRNA, respectively, were amplified by PCR with primers containing T7 promoter sequences. The PCR products were used as templates for *in vitro* transcription with the MEGAscript Kit (Ambion) according to the manufacturer's instructions. After purification of the RNA products by LiCl precipitation, annealing of RNA strands was performed by heating the sample to 65°C for 30 min and subsequent slow (4 h) cooling to room temperature. RNAi treatment was performed by incubating 1×10^6^ cells with a mixture of 10 µg each of the two dsRNA probes for up to 8 days with changes of medium containing fresh dsRNA at days 3 and 5. Cells were harvested at different time points and processed for western blot, immunofluorescence staining or FACS analysis.

### Western blot analysis

For protein expression analyses whole cell lysates were prepared from S2 cells by harvesting the cells into RIPA buffer (50 mM Tris-HCl pH 8, 150 mM NaCl, 1% NP-40, 0.5% deoxycholate, 0.1% SDS, 10 mM NaF, 0.5 mM EDTA, 10% glycerol). Samples were frozen and thawed and subsequently incubated with 25 U/ml benzonase (Novagen) for 30 min at room temperature in the presence of protease inhibitors to release chromatin-bound proteins. Extracts were cleared by centrifugation at 14 000 rpm for 10 min and aliquots (60 µg total protein) were electrophoresed in 6% and 12% SDS-polyacrylamide gels. Separated proteins were blotted and incubated with the following primary antibodies: anti-CHD1pep 1∶2000 (raised in rabbit against a mixture of two peptides corresponding to amino acids 12–26 and 1805–1819, respectively, of the CHD1 sequence; Figure S1A), anti-CID (Abcam) 1∶3000, anti-ISWI 1∶2000 and anti-CAF1 p55 1∶24 000 (gifts of Dr. J.T. Kadonaga). As a secondary antibody peroxidase-conjugated anti-rabbit IgG (GE Healthcare) at 1∶10 000 was used. Detection of antigen-antibody complexes was performed with the ECL Plus system (GE Healthcare). Band intensities were quantified using Adobe Photoshop CS3.

### Micrococcal nuclease treatment and immunoprecipitation

Preparation of mononucleosomal fractions was performed according to Wysocka *et al*. [34]. Briefly, 4×10^8^ S2 cells were collected, washed with phosphate-buffered saline (PBS) and resuspended at 4×10^7^ cells/ml in buffer A (10 mM Hepes/KOH pH 7.9, 10 mM KCl, 1.5 mM MgCl_2_, 0.34 M sucrose, 10% glycerol, 1 mM DTT, protease inhibitor cocktail [Roche]). After addition of Triton X-100 to 0.1% final concentration, cells were homogenized and centrifuged for 5 min at 1300×g. The nuclear pellet was washed with 1 ml buffer A and subsequently lysed in 1 ml of buffer B (3 mM EDTA, 0.2 mM EGTA, 1 mM DTT, protease inhibitor cocktail). Nuclei were centrifuged for 5 min at 1700×g at 4°C, and the insoluble chromatin pellet was resuspended in 0.2 ml buffer M (10 mM Tris/HCl, pH 7.6, 10 mM KCl, 1.5 mM CaCl_2_; prewarmed to 25°C). 1U of micrococcal nuclease (Sigma) was added and digestion was performed for 15 min at 25°C. The reaction was stopped by addition of 1 mM EGTA, and solubilized chromatin was separated from insoluble components by centrifugation at 1700×g for 5 min. An aliquot of the supernatant was deproteinized by Proteinase K digestion and phenol-chloroform extraction. DNA was precipitated, redissolved in 5 µl gel loading buffer and electrophoresed on a 1% agarose gel to check for predominant release of mononucleosomes. The remainder of the supernatant was adjusted to 0.5 ml with buffer C (15 mM Tris/HCl, pH 7.6, 150 mM NaCl, 1 mM EDTA, 1 mM DTT, protease inhibitor cocktail) and incubated over night at 4°C with either anti-GFP or anti-CHD1 antibodies preadsorbed to protein A sepharose beads. Immunoprecipitates were washed with buffer C and eluted by boiling the beads in 2x SDS sample buffer for 5 min. Aliquots of input, supernatant and eluates were subjected to immunoblot analysis.

### Preparation of nuclear extracts

S2 control cells and S2 cells treated for 6 days with dsRNA targeting CHD1 (7×10^7^ cells each) were used to prepare whole cell lysate, nuclei, high-salt nuclear extract and chromatin core fractions exactly as described by Perpelescu *et al*. [21]. Aliquots of each cellular fraction were subjected to SDS PAGE and immunoblotting.

### Immunofluorescence microscopy

S2 cells were harvested and then allowed to settle on cover slips for 1 h. Attached cells were washed with PBS, fixed with 3.7% formaldehyde/0.3% Triton X-100 in PBS for 12 min and subsequently blocked with 1% BSA in PBS. Some cells were treated with 0.1% Triton X-100 for 2 min prior to fixation in order to reduce the amount of soluble antigen. To generate spreads of mitotic chromosomes S2 cells were incubated in 0.05 µg/ml colcemid (Gibco) for 6 h before harvesting. Cells were resuspended in cold 75 mM KCl for 10 min and subsequently centrifuged for 5 min at 4°C. Cells were resuspended in 75 mM KCl, centrifuged onto 8-well poly-lysine coated glass slides and fixed with 5% formaldehyde/0.3% Triton-X-100 in PBS for 10 min. Blocking was in 1% BSA/PBS for 1 h at room temperature.

Primary antibody incubation was performed in 1% BSA/PBS overnight at 4°C; secondary antibody incubation was carried out at room temperature for 1 hour. Cells were repeatedly washed with PBS, DNA was counterstained with DAPI and cells were mounted in Vectashield (Vector Laboratories, Inc.). *Drosophila* embryos were dechorionated and fixed in methanol as previously described [35]. For immunostaining, embryos were rehydrated overnight in 0.15% TBST (50 mM Tris-HCl pH 8, 150 mM NaCl, 0.15% Triton X-100). They were then incubated with primary antibodies in 0.15% TBST for 12 h at 4°C and washed three times (20 min each) in 0.3% TBST before staining with the secondary antibodies for 12 h at 4°C. Embryos were subsequently rinsed three times (20 min each) in 0.3% TBST and incubated for 2 h in 2 mg/ml RNase A solution at 37°C. Following a 10 min wash in 0.3% TBST, embryos were incubated at room temperature for 45 min with propidium iodide (5 µg/ml in 0.15% TBST) or 10 min with DAPI (0.5 µg/ml in 0.15% TBST). Finally, embryos were washed in 0.15% TBST several times and mounted in Vectashield (Vector Laboratories, Inc.).

The following primary antibodies were used: Mouse monoclonal anti-α-tubulin (1∶500; Sigma), mouse monoclonal anti-GFP antibody (1∶200 and 1∶500; Millipore), rabbit polyclonal anti-CID antibody (1∶50 and 1∶300; Abcam), rabbit polyclonal anti-CID antibody (1∶500; gift of Dr. S. Henikoff), rabbit polyclonal anti-CENP-C antibody (1∶5000; gift of Dr. Ch. Lehner), rabbit polyclonal anti-CHD1 antibody (1∶50 and 1∶200; gift of Dr. R. Perry), rabbit polyclonal anti-CHD1pep antibody (1∶250) [Note: the specificity of both anti-CHD1 antibodies for CHD1 was confirmed by immunostaining of CHD1-deficient embryos laid by *Chd1*-null females and of CHD1-depleted S2 cells (Figure S1B and C)], mouse anti-Flag antibody (1∶500; Sigma), rabbit polyclonal anti-BubRI antibody (1∶3000; gift of Dr. C. Sunkel). Appropriate secondary antibodies conjugated with Alexa 350, 488 or 594 (Molecular Probes) were used at 1∶1000.

### Confocal microscopy

Images were taken on a confocal laser scanning microscope (Axiovert 100 LSM510, Carl Zeiss or SP5, Leica) equipped with a 63×/1.40 oil immersion objective. Z series of optical sections were obtained and projected along the z axis to obtain a general view of the specimen. Images were processed using LSM Image Browser (version 4.2) software, ImageJ (version 1.43f) and Adobe Photoshop CS3.

### Quantification of imaging data

Quantification of pixel intensities was performed using ImageJ (version 1.43f) software. To determine CenH3^CID^ fluorescence signal intensities in S2 cells, z stacks were generated of 8 to 10 optical sections in the green channel using maximum intensity projection mode. For CenH3^CID^ intensity measurements of stage 4 embryos, z stacks were generated of 3 to 4 optical sections in the green channel using maximum intensity projection mode. Nuclear regions were selected manually and multiplied with a binary mask of the respective ROIs to obtain the mean centromeric fluorescence intensity. For the quantification of CenH3^CID^ signals in *Drosophila* embryos the absolute centromeric fluorescence intensities per ROI were calculated by multiplying the background corrected mean centromeric pixel intensities with the number of evaluated pixels within the ROI. The obtained values were normalized against the number of centromeric foci. Results from mutant embryos and RNAi-treated S2 cells, respectively, were normalized against wild-type values.

## Supporting Information

Figure S1 Characterization of CHD1 antibodies. A) Schematic representation of the CHD1 protein sequence (top). Polyclonal antibodies were raised in rabbits against a mixture of two peptides corresponding to N-terminal (aa 12–26) and C-terminal (aa 1805–1819) sequences, respectively, of *Drosophila* CHD1. The positions of the peptides are indicated. Peptide-specific antibodies were affinity purified and tested by immunoblotting (bottom). Aliquots of purified recombinant CHD1 (Flag-CHD1), S2 whole cell extract and embryonic extract were loaded onto a 6% SDS polyacrylamide gel, blotted and incubated with the antibodies at 1∶1000 dilution. Signal detection was performed using ECL PLUS reagent (GE Healthcare). B) Anti-CHD1 (top) and anti-CHD1pep (bottom) antibodies (green) were incubated with *Chd1*-deficient haploid blastoderm embryos to test for unspecific cross-reactions. DNA was counterstained with DAPI (red). Weak background staining was observed in the cytoplasm, whereas nuclei were devoid of signal. C) Immunostaining of S2 cells with antibodies against CHD1 (green) as described in B). Cells were either incubated with dsRNA targeting CHD1 (CHD1 RNAi) or water (control) for 6 days before fixation and staining. DNA was visualized with DAPI (blue). D) S2 cells were transiently transfected with Flag-CHD1 and stained with antibodies against Flag (red) and CHD1 (green). DNA was counterstained with DAPI (blue). Flag-CHD1 expressing cells (arrowheads) show stronger anti-CHD1 signals than cells in which Flag-CHD1 is not detectable (asterisks).
(2.98 MB TIF)

Figure S2 CHD1 does not colocalize with centromeres in *Drosophila* S2 cells. A) S2 cells stably expressing EGFP-tagged CenH3^CID^ were treated (bottom panels) or not treated (top panels) with Triton X-100 before fixation to reduce the amounts of soluble protein. Cells were stained with anti-CHD1 peptide antibodies (red) and anti-GFP (green) antibodies to detect CenH3^CID^. DNA is shown in blue. CHD1 displays nuclear staining during interphase and redistributes to the cytoplasm during mitosis. Colocalization of CenH3^CID^ and CHD1 was never observed. B) S2 cells stably expressing Flag-tagged CHD1 were stained with anti-Flag (red) and anti-CenH3^CID^ (green) antibodies. Colocalization of CenH3^CID^ and CHD1 was never observed.
(5.16 MB TIF)

Figure S3 Depletion of CENP-C in S2 cells results in loss of CenH3^CID^ from centromeres. A) S2 cells were treated with dsRNA targeting CENP-C for 4 days. Whole cell extracts from control and RNAi cells were subjected to immunoblotting with antibodies against CENP-C (top) and actin (bottom). CENP-C protein levels were reduced to undetectable amounts. B) CENP-C RNAi treated and control cells were stained with anti-CID antibodies (green) and DAPI to visualize DNA (red). Stainings were performed in cells at day 4 of RNAi treatment. C) Signal intensities of centromeric foci after staining with anti-CenH3^CID^ antibodies were quantified as described in Materials and Methods. Error bars denote standard deviations of signals obtained from 148 untreated and 115 RNAi-treated nuclei, respectively, after 4 days of dsRNA incubation.
(2.45 MB TIF)
