# Supplementary figures and images for: CenH3/CID Incorporation Is Not Dependent on the Chromatin Assembly Factor CHD1 in Drosophila
Source: PLoS One. 2010 Apr 9;5(4):e10120. doi: 10.1371/journal.pone.0010120 (PMC2852906; doi:10.1371/journal.pone.0010120)

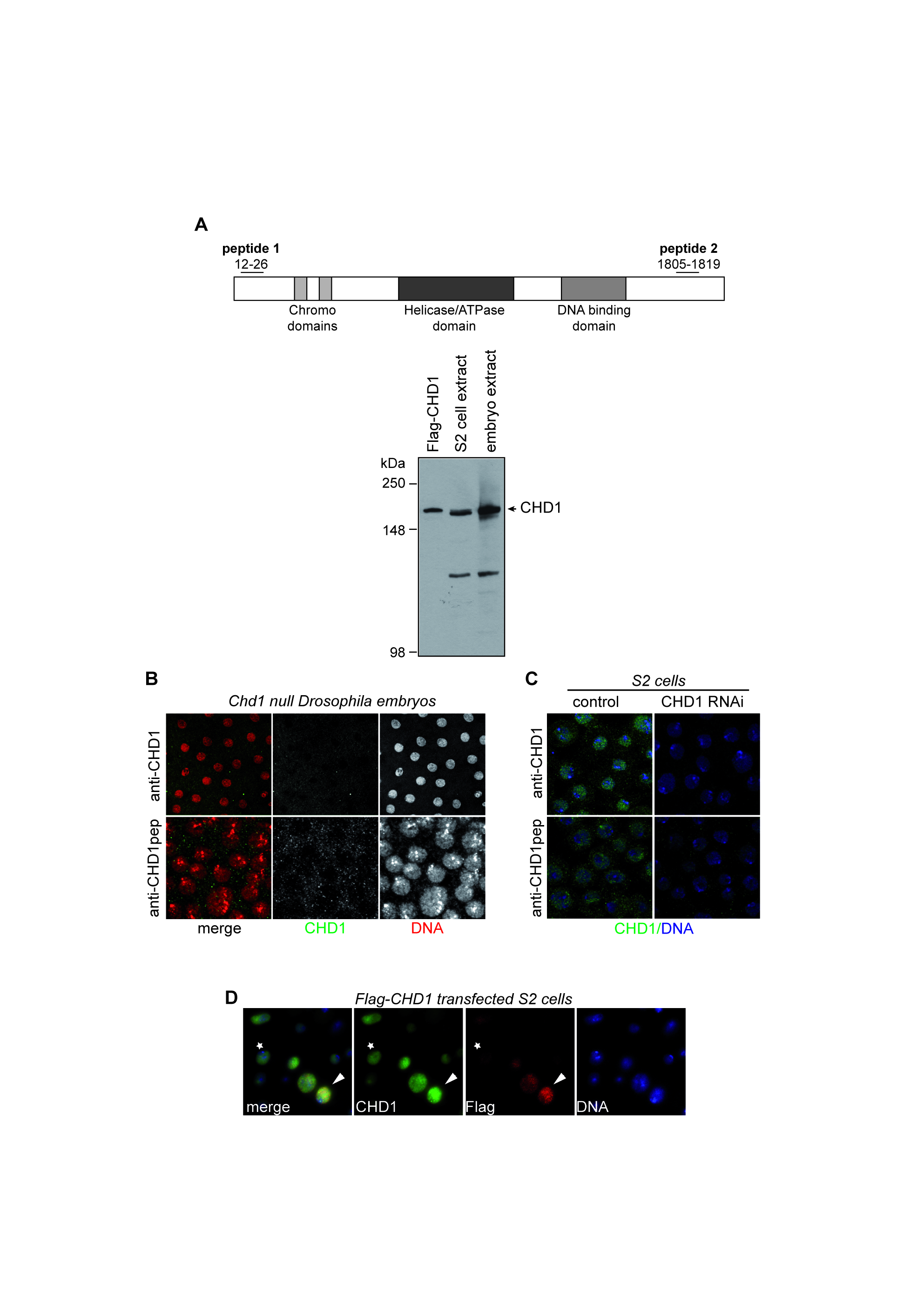

Supplement: Figure S1 — Characterization of CHD1 antibodies. A) Schematic representation of the CHD1 protein sequence (top). Polyclonal antibodies were raised in rabbits against a mixture of two peptides corresponding to N-terminal (aa 12–26) and C-terminal (aa 1805–1819) sequences, respectively, of Drosophila CHD1. The positions of the peptides are indicated. Peptide-specific antibodies were affinity purified and tested by immunoblotting (bottom). Aliquots of purified recombinant CHD1 (Flag-CHD1), S2 whole cell extract and embryonic extract were loaded onto a 6% SDS polyacrylamide gel, blotted and incubated with the antibodies at 1∶1000 dilution. Signal detection was performed using ECL PLUS reagent (GE Healthcare). B) Anti-CHD1 (top) and anti-CHD1pep (bottom) antibodies (green) were incubated with Chd1-deficient haploid blastoderm embryos to test for unspecific cross-reactions. DNA was counterstained with DAPI (red). Weak background staining was observed in the cytoplasm, whereas nuclei were devoid of signal. C) Immunostaining of S2 cells with antibodies against CHD1 (green) as described in B). Cells were either incubated with dsRNA targeting CHD1 (CHD1 RNAi) or water (control) for 6 days before fixation and staining. DNA was visualized with DAPI (blue). D) S2 cells were transiently transfected with Flag-CHD1 and stained with antibodies against Flag (red) and CHD1 (green). DNA was counterstained with DAPI (blue). Flag-CHD1 expressing cells (arrowheads) show stronger anti-CHD1 signals than cells in which Flag-CHD1 is not detectable (asterisks). (2.98 MB TIF) [file pone.0010120.s001.tif]

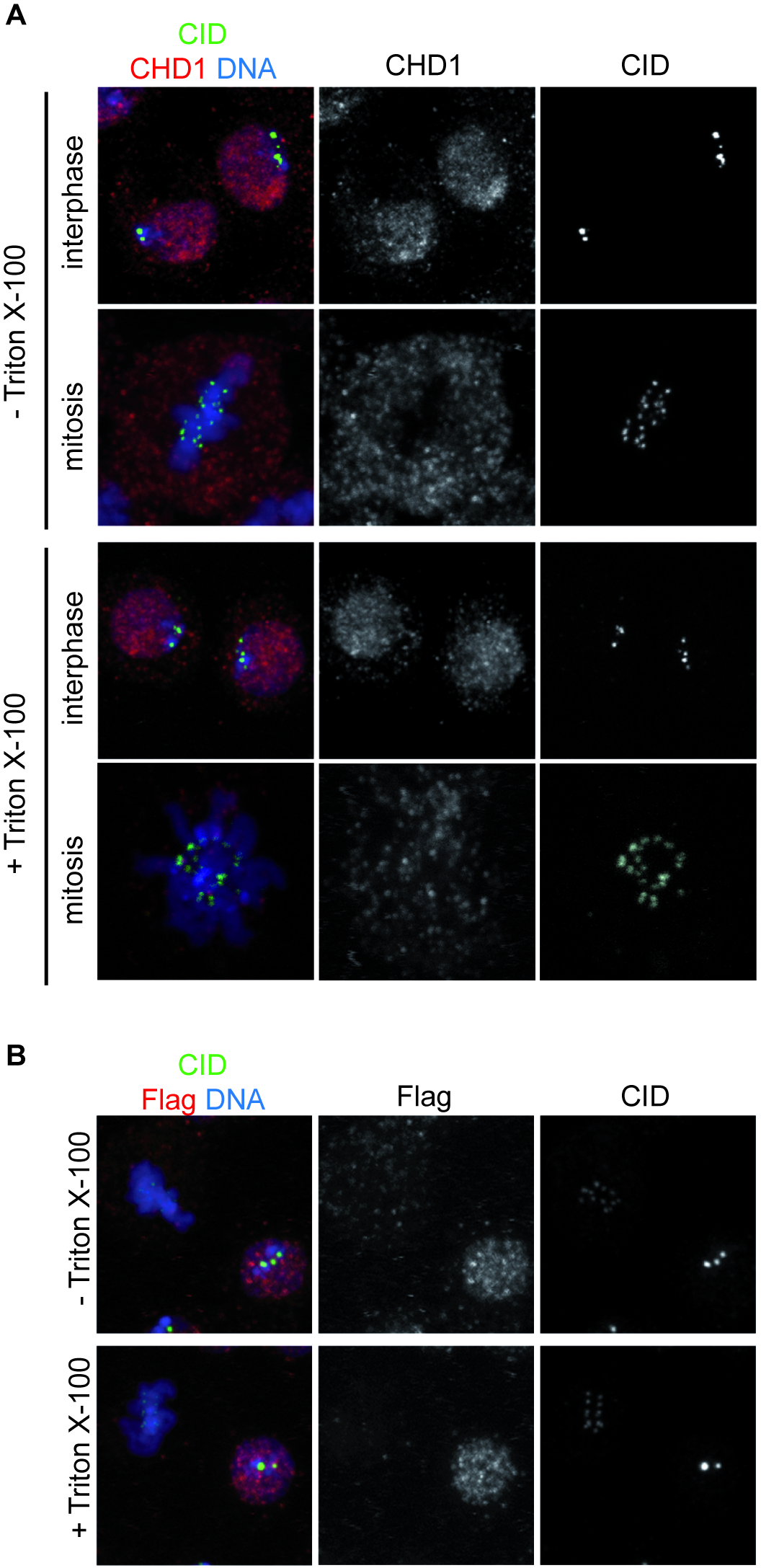

Supplement: Figure S2 — CHD1 does not colocalize with centromeres in Drosophila S2 cells. A) S2 cells stably expressing EGFP-tagged CenH3CID were treated (bottom panels) or not treated (top panels) with Triton X-100 before fixation to reduce the amounts of soluble protein. Cells were stained with anti-CHD1 peptide antibodies (red) and anti-GFP (green) antibodies to detect CenH3CID. DNA is shown in blue. CHD1 displays nuclear staining during interphase and redistributes to the cytoplasm during mitosis. Colocalization of CenH3CID and CHD1 was never observed. B) S2 cells stably expressing Flag-tagged CHD1 were stained with anti-Flag (red) and anti-CenH3CID (green) antibodies. Colocalization of CenH3CID and CHD1 was never observed. (5.16 MB TIF) [file pone.0010120.s002.tif]

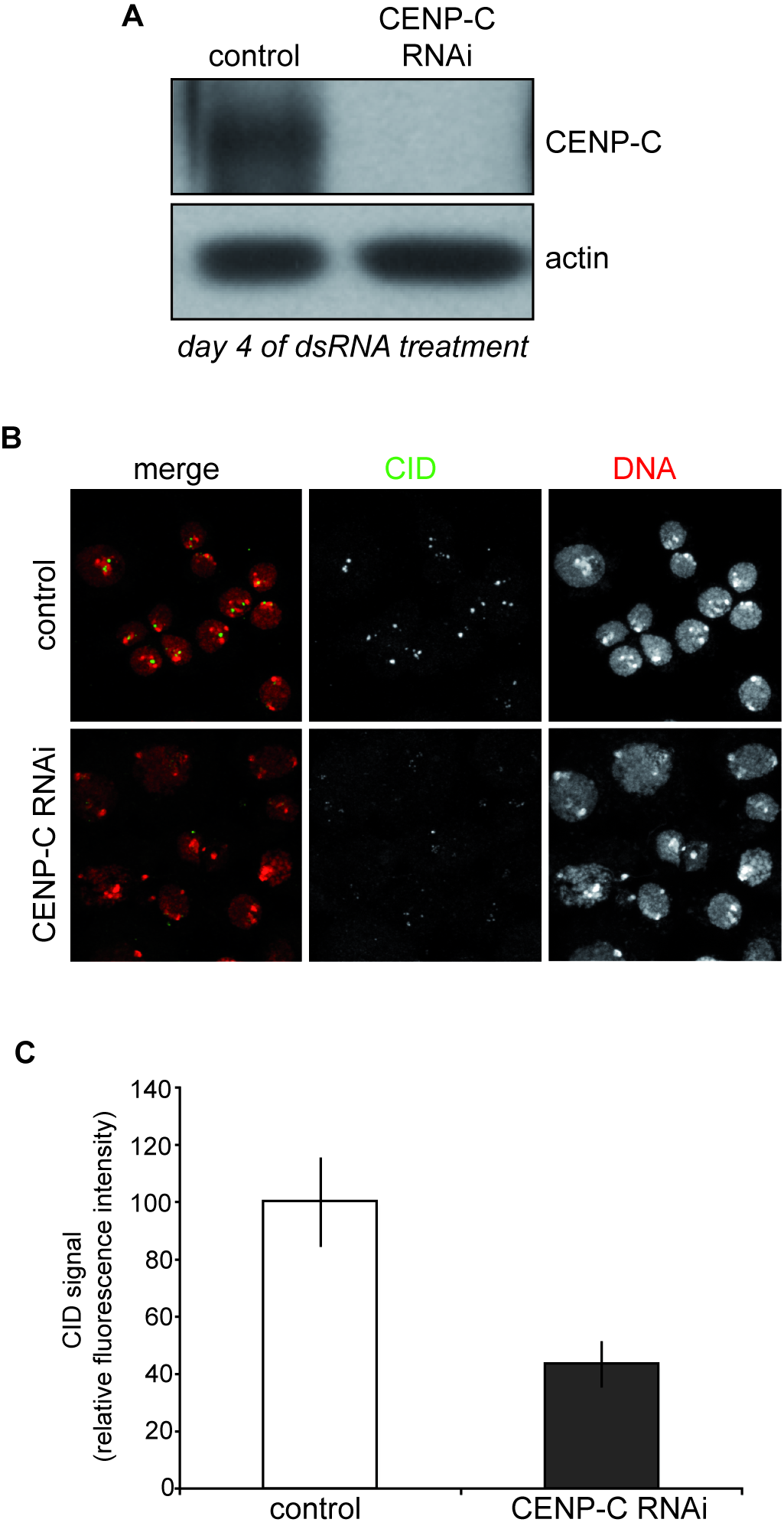

Supplement: Figure S3 — Depletion of CENP-C in S2 cells results in loss of CenH3CID from centromeres. A) S2 cells were treated with dsRNA targeting CENP-C for 4 days. Whole cell extracts from control and RNAi cells were subjected to immunoblotting with antibodies against CENP-C (top) and actin (bottom). CENP-C protein levels were reduced to undetectable amounts. B) CENP-C RNAi treated and control cells were stained with anti-CID antibodies (green) and DAPI to visualize DNA (red). Stainings were performed in cells at day 4 of RNAi treatment. C) Signal intensities of centromeric foci after staining with anti-CenH3CID antibodies were quantified as described in Materials and Methods. Error bars denote standard deviations of signals obtained from 148 untreated and 115 RNAi-treated nuclei, respectively, after 4 days of dsRNA incubation. (2.45 MB TIF) [file pone.0010120.s003.tif]
